# Long non-coding RNA (LncRNA) SNHG7/ Eukaryotic translation initiation factor 4 gamma 2 (EIF4G2) involves in the malignant events of ovarian cancer cells with paclitaxel resistant

**DOI:** 10.1080/21655979.2021.1999555

**Published:** 2021-12-01

**Authors:** Jin Zhang, Rui Zhang, Yongju Ye

**Affiliations:** a Department of Obstetrics and Gynecology, Beijing Shijitan Hospital, Capital Medical University, Beijing, China; b Department of Obstetrics and Gynecology, Lishui Hospital of Traditional Chinese Medicine, Lishui, Zhejiang, China

**Keywords:** LncRNA SNHG7, EIF4G2, paclitaxel resistance, ovarian cancer

## Abstract

LncRNA SNHG7 shows a strong relationship with malignant behavior of cancer cells and poor clinical outcome in cancer. The resistance of ovarian cancer for Paclitaxel seriously limits the clinical efficacy in chemotherapy for ovarian cancer patients. In this study, we investigated whether lncRNA SNHG7 was involved in Paclitaxel sensitivity of ovarian cancer as well as the underlying mechanism regulating the behavior of ovarian cancer cells with Paclitaxel resistance. The experiment results of wound healing and transwell showed that in paclitaxel-resistant ovarian cancer cells, transfection with siRNA-SNHG7 in ovarian cancer cells reduced cell migration and invasion. And cell cycle was observed by means of Flow cytometry. RNA immunoprecipitation assay was performed to analyze the interaction of lncRNA SNHG7 and EIF4G2. Overexpression of EIF4G2 by transfection with Ov- EIF4G2 plasmids efficiently blocked the changes of migration and invasion, as well as G0/1 arrest caused by lncRNA SNHG7 silencing. Taken together, these results demonstrated that lncRNA SNHG7 could affect the degradation of EIF4G2 to regulate the sensitivity of ovarian cancer to Paclitaxel, inhibit cell viability, migration, and invasion. The interaction of lncRNA SNHG7 and EIF4G2 plays an important role in the migrative and invasive activity and Paclitaxel resistance of ovarian cancer cells.

## Introduction

Ovarian cancer is a malignant tumor found only in the female reproductive system with high morbidity and mortality [1]. Currently, treatment available for ovarian cancer focuses on paclitaxel and platinum. Despite the fact that a majority of patients have a good response to initial chemotherapy, almost all patients experience multiple-disease recurrences [2,3]. LncRNAs play vital roles in biological function of cancer cells, which is involved in regulating the activity of the corresponding protein by binding to a specific protein [4–7]. The cellular function of lncRNAs begins in part with the cellular localization of lncRNAs: lncRNAs rich in the nucleus are primarily responsible for nuclear structure, chromatin interactions, regulation of transcription, and processing of RNA functions, while lncRNAs in the cytoplasm can regulate mRNA stability or translation and affect cellular signaling cascades [4,8–11]. LncRNA SNHG7 is a typical molecule that plays the role of proto-oncogene effect. It is highly expressed in a variety of tumors and plays a role in promoting the development of malignant tumor phenotypes [12,13].

EIF4G2 has been implicated in the development and progression of some tumors. A study found that eIF4G2 expression was high in primary diffuse large B cell lymphoma cells [14]. Downregulation of eIF4G2 expression by siRNA can inhibit translation, affect cell viability and colony formation ability, and induce cell senescence. Additionally, in ovarian cancer cells, EIF4G2 is implicated in paclitaxel resistance [15]. To fully probe the effect of lncRNA SNHG7, this paper showed that lncRNA SNHG7 could combine with EIF4G2 through Starbase database (http://starbase.sysu.edu.cn/) and RIPseq database. We predicted that there may exist an association between lncRNA SNHG7 and EIF4G2, which could participate in the paclitaxel-resistance of ovarian cancer cell. Therefore, this paper intended to investigate the role of lncRNA SNHG7 in ovarian cancer and analyze the relationship between lncRNA SNHG7/EIF4G2 and paclitaxel resistance, thereby elucidating the mechanism of lncRNA SNHG7/EIF4G2 in paclitaxel-resistance of ovarian cancer cell.

## Method

### Cell culture

SKOV3, heyA8 and Paclitaxel (PTX) resistant ovarian cancer cell line SKOV3/PTX and heyA8-PTX cells were all from China Center for Type Culture Collection. The base medium required for cell culture was DMEM/F12 containing 10% fetal bovine serum 1 × 10^5^ U/L penicillin and 100 mg/L streptomycin. Cells were incubated in a suitable incubator of 37°C with 5% CO2.

### Quantitative reverse transcription PCR (RT-qPCR)

Extraction of total RNA employed Trizol method, and synthesis of cDNA was carried out by reverse transcription. The PCR primers were generated by Shanghai Sangon Bioengineering Co., Ltd. GAPDH acted as an internal reference. LncRNA SNHG7 or EIF4G mRNA level was determined by RT-PCR (Light Cycler@480, Roche Company). The 2 ^−ΔΔCT^ method was applied to figure out the RNA levels [16].

### Cell Counting Kit-8 (CCK8) assay

Cell viability was measured as per the instructions of CCK-8 kit (MSK, Wuhan, China). The logarithmic growth phase cells were seeded in 96-well plates (100 μL/well) and cultured for 0 and 24 h in an incubator at 37°C with 5% CO2. Then, 10 μL CCK-8 solution was incubated with the cells in each well for 2 h. The absorbance of each well at 450 nm was examined with the application of a microplate analyzer.

### Western blotting

Protein extraction was carried out by RIPA Lysis Buffer for 15 min. Then, the centrifugation to cell lysis was conducted at 12000 r/min for 15 min at a centrifugation radius of 10 cm. Protein concentration was assessed by means of BCA method. In the following experiment, proteins were electrophoresed using sodium dodecyl sulfate-polyacrylamide gel electrophoresis (SDS-PAGE) and then transferred to PVDF membranes, which then was soaked into 5% skimmed milk powder for 1 h. The following incubation of protein with the primary or secondary antibodies (Abcam, England) was performed at 4°C overnight or at room temperature for 1 h, respectively. Image analysis software Image J was used to detect the gray values of protein bands.

### Transwell assay

After transfection, cell suspension of each group was added 200 μL to the upper chamber of each Transwell chamber (Transwell BD Matrigel (FN), Costar, USA). Then, 600 μL DMEM/F12 medium with 10% fetal bovine serum was added to the lower chamber. The Transwell chamber was placed in a 24-well plate and grown in an incubator at 37°C with a 5% CO_2_ in air atmosphere. 48 h later, Transwell chamber was taken out, followed by wash with PBS for 3 times. 10 minutes fixation (4% paraformaldehyde) and 20 minutes staining (0.1% crystal violet) were carried out in sequence. Four fields were randomly selected under an inverted microscope to calculate the number of transmembrane cells.

### Wound healing assay

The scratch test plug-in was placed in the 24-well plate, and ovarian cancer cells at the logarithmic growth phase was adjusted to 5 × 10^5^ cells and seeded into the 24-well plate. The scratch test plug-in was carefully removed the next day. Replacement of used culture medium was performed employing fresh complete medium. The culture was continued for 24 h and the photo was taken.

### RIP assay

The RIP experiment was performed according to the instruction manual of RNA Immunoprecipitation Kit (SIGMA). The general experimental steps are as follows: EIF4G2 antibody (abcam, England) or mouse IgG were incubated with magnetic beads for 2 h. Cell lysates were then cultured overnight together with magnetic beads, the proteins in the RNA-protein complex were washed, digested, and the RNA was purified for real-time PCR detection.

### Statistical analysis

GraphPad Prism 7.0 was used to analyze the experimental data among different groups through One-way ANOVA analysis, followed by turkey’s test and draw the relevant pictures for the analysis results of the experimental data. It was judged to be statistically significant when P was less than 0.05.

## Result

### LncRNA SNHG7 silencing suppressed cell viability

**Figure 1.**
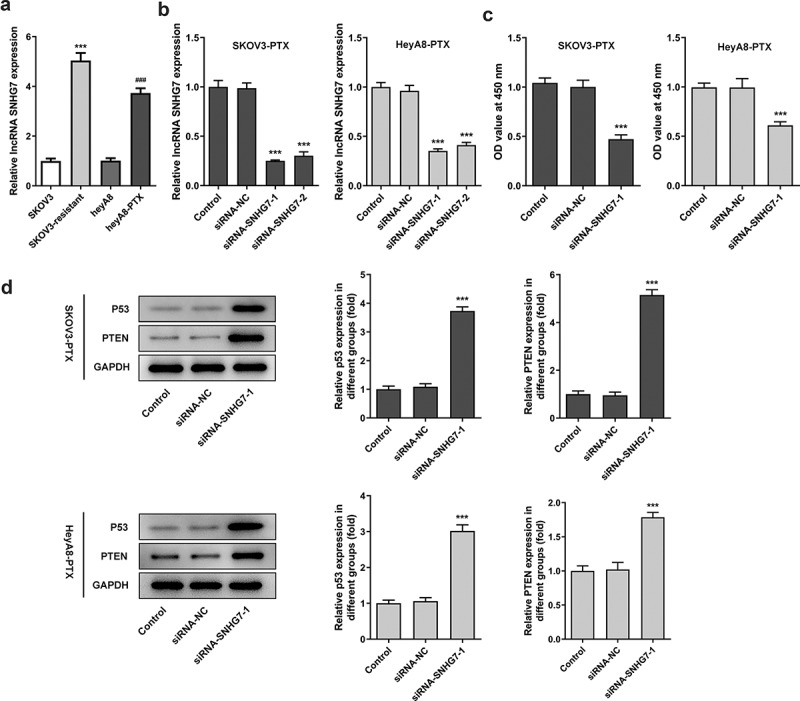
LncRNA SNHG7 silencing reduced cell viability and increased the protein levels of p53 and PTEN. (a) The expression of lncRNA SNHG7 in SKOV3, HeyA8, SKOV3-PTX and HeyA8-PTX cells was detected by RT-qPCR. ***P < 0.001 versus SKOV3, ^###^P < 0.001 versus HeyA8. (b) The interference plasmid of lncRNA SNHG7 was constructed, and the expression of lncRNA SNHG7 was detected by RT-qPCR. ***P < 0.001 versus siRNA-NC. (c) Cell viability was detected by CCK8 and OD value was calculated. ***P < 0.001 versus siRNA-NC. (d) The expressions of P53 and PTEN were detected by Western blot. ***P < 0.001 versus siRNA-NC

To analyze the role of lncRNA SNHG7 in ovarian cancer cells with resistance to PTX, qRT-PCR was in detection of the expression of lncRNA SNHG7 in SKOV3 and heyA8, as well as their corresponding drug-resistant cell lines (Figure 1a). The results showed that lncRNA SNHG7 expression was significantly increased in ovarian cancer cell lines SKOV3 and heyA8 with resistance to paclitaxel PTX compared with corresponding cell lines without drug resistance (Figure 1a). In this study, siRNA-SNHG7 plasmids were constructed for transfection of SKOV3-PTX or heyA8-PTX cells to interfere with the expression of lncRNA SNHG7 in SKOV3-PTX cells and heyA8-PTX cells. The silencing effect of siRNA-SNHG7 was detected by qRT-PCR. It was shown that siRNA-SNHG7 markedly decreased lncRNA SNHG7 levels in SKOV3-PTX cells and heyA8 cells (Figure 1b). The silencing effects of siRNA-SNHG7-1 seemed to be superior to siRNA-SNHG7-2. Thus, siRNA-SNHG7-1 was chosen to perform subsequent experiment. Next, CCK8 assay was used to observe the effect of lncRNA SNHG7 silencing on the viability of SKOV3-PTX or heyA8-PTX cells. Compared with the siRNA-NC group, SKOV3-PTX or heyA8-PTX cells viability was greatly declined (Figure 1c). Next, Western blot was used to detect the expression level of PTEN and p53 in SKOV3-PTX or heyA8-PTX cells after lncRNA SNHG7 silencing. Increased PTEN and p53 levels were observed in both KOV3-PTX and heyA8-PTX cells after lncRNA SNHG7 silencing (Figure 1d).

### lncRNA SNHG7 silencing suppressed cell migration and invasion

**Figure 2.**
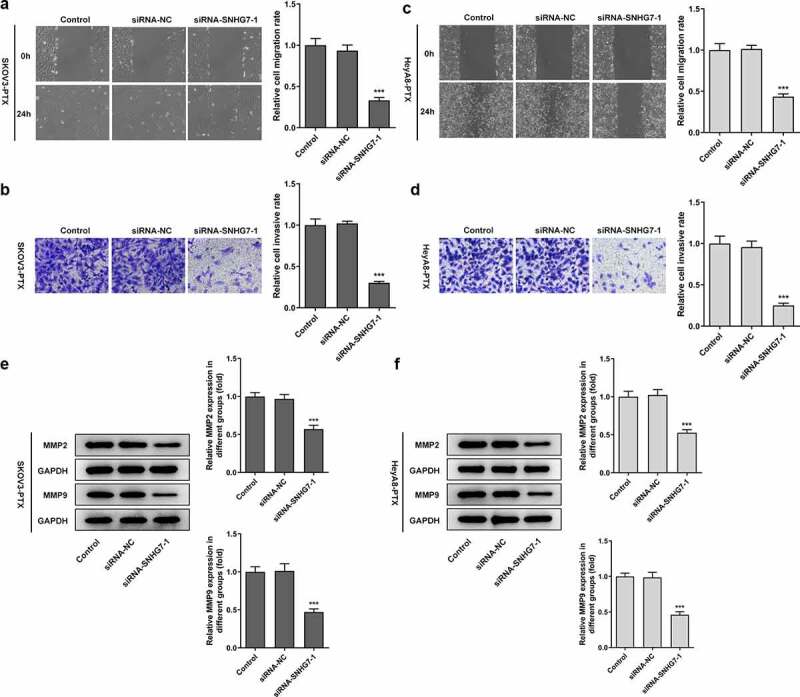
After interference by siRNA-SNHG7, the invasion and migration of SKOV3-PTX and HEYA8-PTX cells were inhibited. (a-b) The invasiveness and migration of cells were detected by Transwell assay and Wound healing assay. ***P < 0.001 versus siRNA-NC. (c-d) Total cell lysates were immunoblotted with MMP2 or MMP9 antibodies. ***P < 0.001 versus siRNA-NC

To determine the loss function of lncRNA SNHG7, the effects of lncRNA SNHG7 silencing on SKOV3-PTX and heyA8-PTX cells migration and invasion ability were assessed with the adoption of Transwell and wound healing assays. As shown in Figure 2a-b, both SKOV3-PTX and heyA8-PTX with lncRNA SNHG7 silencing exhibited low migration and invasion as relative to siRNA-NC group. Additionally, the expression of MMP2 and MMP9 was significantly inhibited when lncRNA SNHG7 silencing was induced Figure 2c-d.

### LncRNA SNHG7 interference enhanced the sensitivity of SKOV3-PTX and HeyA8-PTX cells to paclitaxel

**Figure 3.**
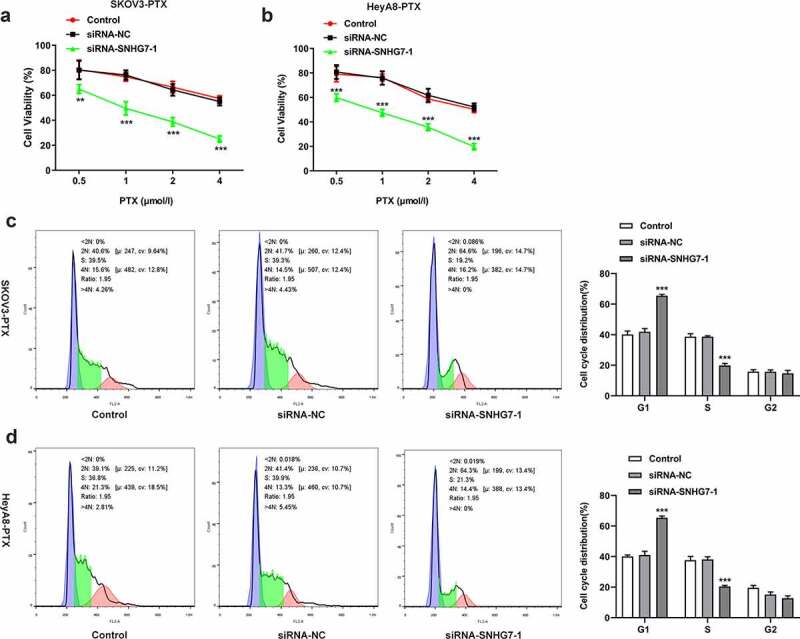
Silencing of lncRNA SNHG7 in PTX-resistant ovarian cancer cells leads to enhanced the sensitivity of SKOV3-PTX or heyA8-PTX to PTX and promotes G1 arrest. (a-b) CCK8 assay was performed. (c-d) Flow cytometry was performed to detect cell cycle. **P < 0.01, ***P < 0.001 versus siRNA-NC

Paclitaxel was reported to be able to increase the apoptosis of paclitaxel-sensitive ovarian cancer cell. However, drug resistance greatly limited success clinical therapy [17]. To determine the role of lncRNA SNHG7 in PTX resistance of ovarian cancer cell, the loss function of lncRNA SNHG7 was evaluated by the induction of lncRNA SNHG7 silencing in SKOV3-PTX and HeyA8-PTX cells. CCK8 assay was used to detect the cell activities of SKOV3-PTX or heyA8-PTX. Gene silencing of lncRNA SNHG7 with shRNA efficiently increased the sensitivity of SKOV3-PTX or heyA8-PTX to PTX (Figure 3a-b). The IC50 value in Control group is 6.979 µmol/L, in SiRNA-NC group is 5.558 µmol/L, SiRNA-SNHG7-1 is 1.050 µmol/L in SKOV3-PTX cells while in heyA8-PTX cells, the IC50 value in Control group is 3.897 µmol/L, in SiRNA-NC group is 4.434 µmol/L, SiRNA-SNHG7-1 is 0.858 µmol/L. LncRNA SNHG7 silencing induced G0/1 arrest not only in SKOV3-PTX but also in heyA8-PTX (Figure 3c-d). These findings indicated that lncRNA SNHG7 acts as a key player in mediating the resistance of ovarian cancer cells for PTX.

### Interference with lncRNA SNHG7 inhibited the expression of EIF4G2 in paclitaxel-resistant ovarian cancer cells viability

**Figure 4.**
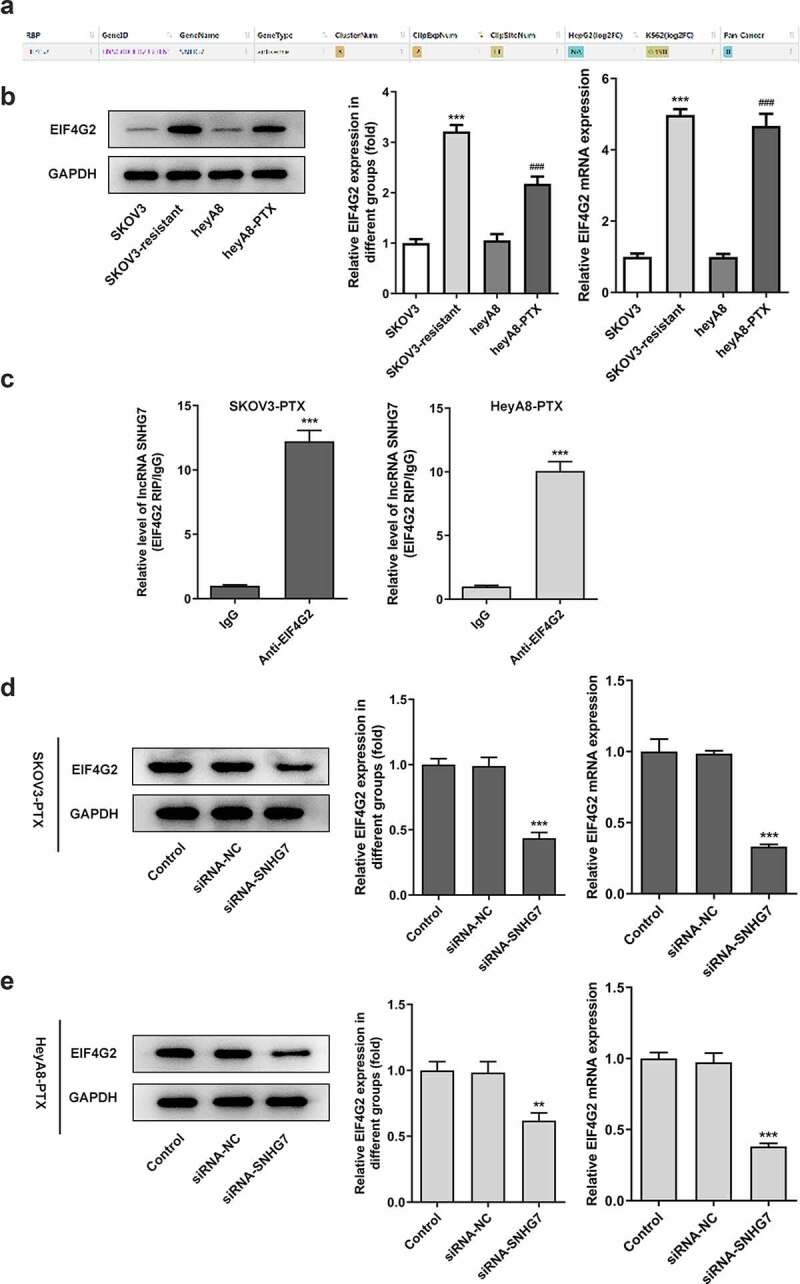
LncRNA SNHG7 interacted with EIF4G2 and lncRNA SNHG7 silencing reduced EIF4G2 levels. (a) A schematic of the putative interacting sites of lncRNA SNHG7 and EIF4G2. (b) Western blot and Quantitative real-time RT-PCR (qPCR) were performed to determine the expression of EIF4G2 in SKOV3, SKOV3-PTX, HeyA8 and HeyA8-PTX cells. ***P < 0.001 versus SKOV3, ^###^P < 0.001 versus HeyA8. (c) RIP assay was performed to analyze the interaction of lncRNA SNHG7 and EIF4G2. ***P < 0.001 versus IgG (d-e) qPCR was performed to determine the expression of EIF4G2. **P < 0.01, ***P < 0.001 versus siRNA-NC

To further explore the mechanism of action of lncRNA SNHG7, Starbase database, and RIPseq database were used to predict whether lncRNA SNHG7 could be combined with eIF4G2 as shown in Figure 4a. The result demonstrated that there might be an interaction between lncRNA SNHG7 and EIF4G2. Therefore, we next investigated whether lncRNA SNHG7 could interact with EIF4G2 and EIF4G2 affects the effects of lncRNA SNHG7 silencing on cell sensitivity for PTX. First, we first detected the expression of EIF4G2 through RT-qPCR and Western blotting among four ovarian cancer cell lines (SKOV3, SKOV3-PTX, heyA8, and heyA8-PTX). EIF4G2 expression showed higher levels in SKOV3-PTX when compared to SKOV3, while presented higher levels in heyA8-PTX when compared to heyA8 (Figure 4b). qPCR results of lncRNA SNHG7 showed that there was massive enrichment of lncRNA SNHG7 in immunoprecipitation formed by Anti-EIF4G2 antibody in SKOV3-PTX cells and heyA8-PTX cells, revealing that EIF4G2 interacted with lncRNA SNHG7 (Figure 4c). Additionally, the targeted inhibition of lncRNA SNHG7 decreased the expression of EIF4G2 (Figure 4d-e). These findings supported that lncRNA SNHG7 might affect EIF4G2 degradation.

### LncRNA SNHG7/EIF4G2 interaction affected paclitaxel-resistant ovarian cancer cells viability

**Figure 5.**
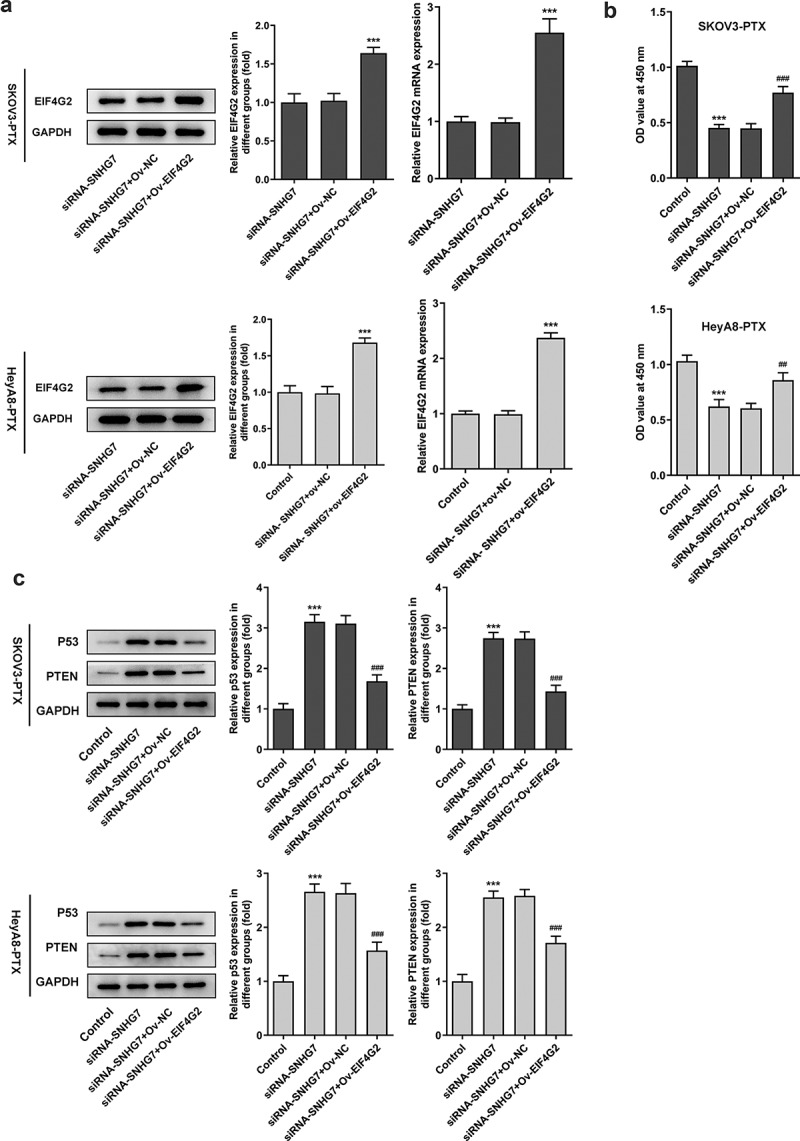
LncRNA SNHG7/EIF4G2 interaction affected viability and the protein levels of SKOV3-PTX or HEYA8-PTX cells. (a) cell viability was detected by CCK8 assay kit. ***P < 0.001 versus siRNA-SNHG7+ Ov-NC. (b-c) Total cell lysates were immunoblotted with p53 or PTEN antibodies. ***P < 0.001 versus Control, ^##^P < 0.01, ^###^P < 0.001 versus siRNA-SNHG7+ Ov-NC

To determine whether the function of lncRNA SNHG7 was relevant to expression of EIF4G2 in paclitaxel-resistant ovarian cancer, we first induced EIF4G2 overexpression through transfecting EIF4G2 overexpression plasmids into SKOV3-PTX or HEYA8-PTX cells (Figure 5a). The overexpression of EIF4G2 markedly abrogated the effects of lncRNA SNHG7 silencing on decreasing cell viability of SKOV3-PTX or heyA8-PTX, and promoting the expression of p53 and PTEN (Figure 5b-c). These results suggested that EIF4G2 affected the effects of lncRNA SNHG7 on cell viability of SKOV3-PTX or HEYA8-PTX.

#### LncRNA SNHG7/EIF4G2 interaction affected paclitaxel-resistant ovarian cancer cells

**Figure 6.**
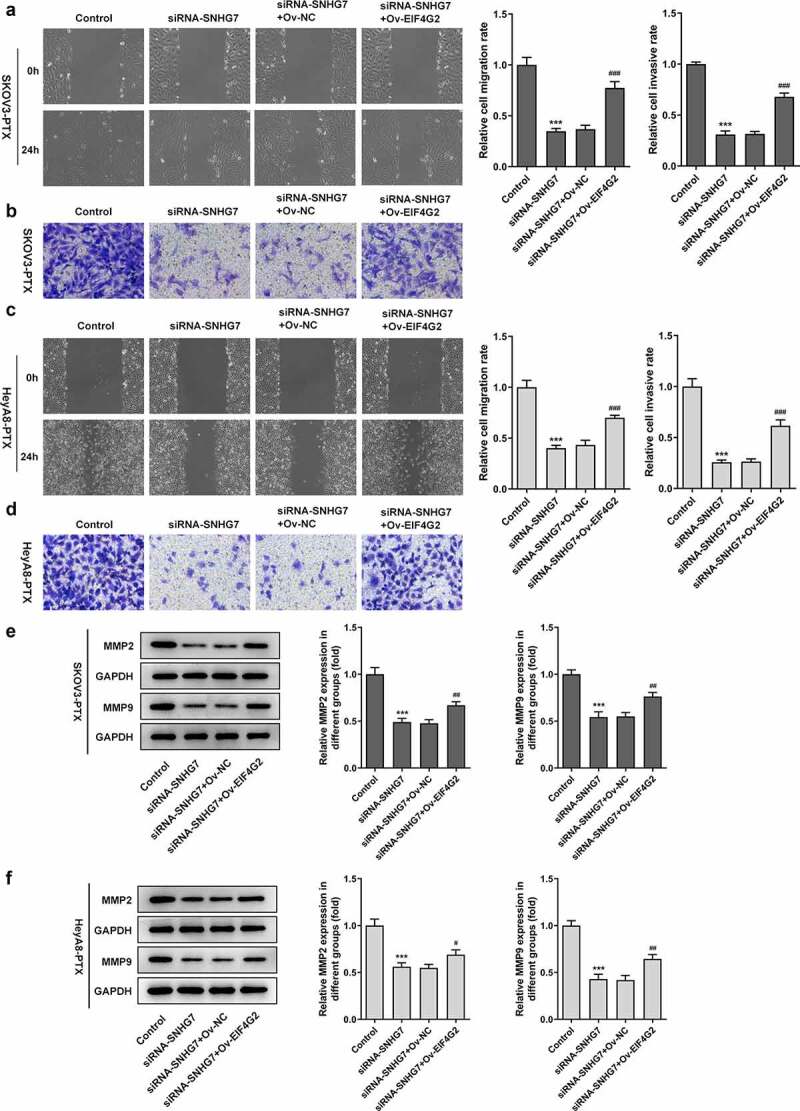
LncRNA SNHG7/EIF4G2 interaction affected invasion and migration of paclitaxel-resistant ovarian cancer cells. (a-b) The migration and invasion of cells were detected by Transwell and Wound healing assay, respectively, as described in the Materials and Methods. (c-d) The expression of MMP2 and 9 was analyzed through western blot assay. ***P < 0.001 versus Control, ^#^P < 0.05, ^##^P < 0.01, ^###^P < 0.001 versus siRNA-SNHG7+ Ov-NC

**Figure 7.**
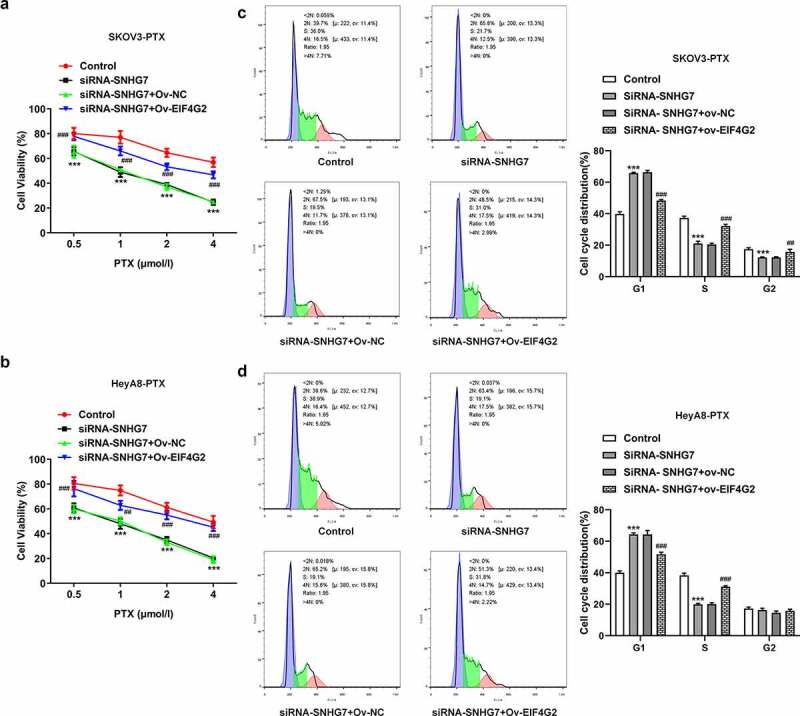
LncRNA SNHG7/EIF4G2 interaction affected cell viability and G1 arrest. (a-b) The cell activity was detected by CCK8 assay in SKOV3-PTX or HEYA8-PTX cells. (c-d) Flow cytometry was used to analyze the cell cycle. **P < 0.001 versus Control, ^##^P < 0.01, ^###^P < 0.001 versus siRNA-SNHG7+ Ov-NC

We next investigated whether EIF4G2 involve cancer metastasis in paclitaxel-resistant ovarian cancer cells. Paclitaxel-resistant ovarian cancer cells not only enhanced invasion and migration (Figure 6a-b) but also increased the expression of MMP2 and 9 in inducing the overexpression of EIF4G2 (Figure 6c-d) when compared to SiRNA- SNHG7 group. The up-regulation of EIF4G2 using ov-EIF4G2 transfection prevented the decreased cell viability by EIF4G2 overexpression in SKOV3-PTX or HEYA8-PTX cells (Figure 7a-b). Furthermore, the overexpression of EIF4G2 reversed the effects of lncRNA SNHG7 silencing on G0/G1 arrest (Figure 7c-d).

## Discussion

Exploring the key molecules involved in drug sensitivity is an important way to reverse chemotherapy resistance in tumors, but the key molecules or mechanisms involved in chemotherapy resistance in ovarian cancer still need to be further elucidated. Paclitaxel-resistance in ovarian cancer greatly hiders the success utilization and therapy of paclitaxel in clinic [17]. Prior studies noted that the involvement of small noncoding RNAs was associated with the mechanism of paclitaxel resistance in ovarian cancer [18–20]. Accordingly, in our work, a series of experiments were carried out to analyze the possible mechanism of paclitaxel-resistance in ovarian cancer. The results showed that when compared to ovarian cancer cell without PTX-resistance, ovarian cancer cell with that express higher levels of lncRNA SNHG7. Our work found that SNHG7/EIF4G2 was able to regulate p53 expression levels and affect G1 arrest. p53 plays a vital role in apoptosis and G1 arrest in ovarian cancer cells [21,22]. We predict that SNHG7/EIF4G2 could be involved in regulating apoptosis via p53, which demands deeper studies to verify it, which is the limit of this study.

LncRNA SNHG7 silencing reduced viability, invasion, and migration of SKOV3-PTX and heyA8-PTX cells. Additionally, low lncRNA SNHG7 expression also leads to reduced proliferation, the migration, and invasion of tumor cells [23–25]. Some researchers reported that this regulatory role of lncRNA SNHG7 was related to the involvement of some signaling pathway containing AKT/mTOR signaling pathway and Wnt/beta-catenin/EMT signaling pathway, or mediated by microRNAs [26–28]. In addition, the down-regulation of lncRNA SNHG7 enhanced the sensitivity of cells to PTX as detected by means of CCK8 assay. This molecular mechanism of lncRNA SNHG7 was considered to be related to EIF4G2 in the present study, which was further confirmed by RIP assay and EIF4G2 overexpression assay. On the other hand, we found that lncRNA SNHG7 silencing induced the low expression of EIF4G2 and the induction of EIF4G2 overexpression markedly reversed the influence of lncRNA SNHG7 silencing on the viability, invasion, and migration of SKOV3-PTX and heyA8-PTX cells. Based on these, we predicted that the interaction of lncRNA SNHG7 and EIF4G2 could affect the degradation of EIF4G2 to affect PTX-sensitivity of SKOV3-PTX or heyA8-PTX and some malignant behavior of these cells. It was reported that protein interacting with LncRNA could play a vital role in stabilizing LncRNA [29,30]. Maybe this interaction could affect the stability of lncRNA SNHG7, which required further investigated. EIF4G2 has been investigated to be associated with the resistance of ovarian cancer to paclitaxel, which was recognized as a potential target for the therapy of PTX-resistant ovarian cancer [15].

### Conclusion

To sum up, we firstly discovered the novel mechanism of lncRNA SNHG7/EIF4G2 to affect the PTX-sensitivity in chemo-resistant ovarian cancer cells, which offers a novel insight to further investigate the molecular mechanism of PTX-resistance in ovarian cancer.

## Limitation

The work reveals that lncRNA SNHG7/EIF4G2 is involved in drug resistance of ovarian cancer cells for PTX in vitro. However, the association of lncRNA SNHG7 and EIF4G2 in the PTX-resistance needs further analysis and exploration in vitro and vivo, which is the limit of this study, which should be made consideration in the future.

## Highlights

1. Targeting lncRNA SNHG7 suppresses the cell viability, migration and invasion of ovarian cancer cells resistant to PTX.

2. LncRNA SNHG7 interacts with EIF4G2.

3. LncRNA SNHG7/ EIF4G2 affects the sensitivity of ovarian cancer cells with PTX -resistance to PTX.

## Novelty statement

The study firstly revealed the novel mechanism of lncRNA SNHG7/EIF4G2 to affect the PTX-sensitivity in chemoresistant ovarian cancer cells, which offers a novel insight to further investigate the molecular mechanism of PTX-resistance in ovarian cancer.
